# Na we go shine: women's wellbeing, agency, and health seeking behaviours in southeastern Nigeria

**DOI:** 10.3389/fgwh.2025.1550817

**Published:** 2025-07-22

**Authors:** Farah M. C. Shroff

**Affiliations:** ^1^ Department of Family Practice, Faculty of Medicine, University of British Columbia, Vancouver, BC, Canada; ^2^ Harvard Health Accelerator, Harvard TH Chan School of Public Health, Boston, MA, United States; ^3^ Health Together (formerly Maternal and Infant Health Canada)—Global Public Health Organization, Vancouver, BC, Canada

**Keywords:** Nigeria, women's health, traditional medicine, work culture, rural

## Abstract

**Objectives:**

Peer-reviewed literature on southeastern Nigerian women's health status is scant. This participatory action research project explored mental and physical health status issues within a sample population of childbearing women in Cross River State.

**Methods:**

We conducted an initial study using the formal chieftaincy channels in villages and learned that those who expressed themselves were primarily men. We found that their concerns differed from those of women. We conducted this study in an attempt to hear from women about their health needs in the context of their lives. Local women carried out face-to-face interviews in their language with childbearing women in their community. We interviewed 70 women from ages 18–45 in 12 villages.

**Results/discussion:**

Most participants had their own farms and grew rice, cassava and yam to feed their families. The majority of participants had not completed elementary school and had given birth to an average of 6 children, 4 of whom survived. Most of the women who were included in this study walked 6–8 h per day to retrieve drinking water. Their young children and babies often accompanied their mothers on these journeys. Participants reported that they had suffered from malaria, diarrhea, anemia, hernia, waist pains, cough, eye problems and continuous headaches. Traditional healers were their first choice for treatment, partly because of physical and financial accessibility and partly because of cultural resonance and positive outcomes.

**Conclusion:**

Despite tremendous social, economic and political barriers, our participants generally reported a strong sense of well-being and had positive outlooks on their lives. We also interviewed 15 traditional healers to enhance the statements made by our female participants regarding their health-seeking behaviors.

## Introduction

Mental and physical health are shaped by structural determinants such as income, education, human rights, and other social factors. Additionally, health seeking behaviors play an important role in influencing overall health outcomes. The constitution of the World Health Organization (WHO) states that “the enjoyment of the highest attainable standard of health is one of the fundamental rights of every human being, without the distinction of race, religion, political belief, economic or social condition.” (1) Dr. Margaret Chan, Director General of the WHO, asserts that women need special attention in health agendas for the following key reasons: women are agents of change and they can lift households and communities out of poverty; their role as caregivers makes them an important resource; and they are susceptible to special health problems and a heightened risk of mortality (2). Interventions that aim to improve women's health must be developed from the outset with an understanding of women's perceptions of their own health issues, and how these are linked to other areas of their lives. In order to achieve this, health planners must engage women in their own communities, encourage expression and commit to listen to what women have to say (3).

With these ideas in mind this study was designed to understand health issues from female perspectives, based on a small group, in southeastern Nigeria. Table 1 provides basic demographic information about Nigeria.

**Table 1:** Nigerian demographics.

Demographic	Value
Population	177,155,754 (4)
Annual Population Growth Rate	2.47% (4)
Population of 0–14 year olds	43.2% (4)
Life Expectancy: Women	53.66 years (4)
Life Expectancy: Men	51.63 years (4)
Total Fertility Rate	5.25 children per woman (4)
Infant Mortality Rate	74.09 per 1,000 live births (4)
Maternal Mortality Rate	630 per 100,000 births (4)
Probability of dying under five (per 1,000 live births, 2012)	124
Human Development Index (HDI)^a^	0.47 (5)
Nigeria's World Ranking (HDI)	153 out of 187 (5)

^a^ The Human Development Index (HDI) provides a composite measure of three dimensions of human development: living a long and healthy life (measured by life expectancy), being educated (measured by adult literacy and enrolment at the primary, secondary and tertiary level) and having a decent standard of living [measured by purchasing power parity (PPP) and income].

Approximately 70% of Nigerians live under the poverty line (4). Cross River, in Southeastern Nigeria, is one of the country's most poverty-stricken states. Based on an agricultural economy, most rural women and men gain their livelihood from farming and trading. Economic development is relatively slow.

There is a growing body of literature that explores health-related issues among Nigerian women. This includes studies of infertility (6), breast cancer (7, 8), cervical cancer (9), sickle cell anemia (10), sexual behavior, contraception (11, 12) and reproductive aspirations (13). Further, studies have been done that involve Nigerian women identifying their health issues and priorities using qualitative methods. For example, Ene-Obong et al. examined the effect of a variety of socioeconomic and cultural factors on health and nutritional outcomes of women of childbearing age, using qualitative and quantitative methods (14). Okafor applied a participatory approach to implement a safe motherhood project, from needs assessment to evaluation (15). There is also literature from other African nations that involve women self-identifying and discussing their health priorities and experiences (16, 17), and from other countries such as China, Ecuador and Brazil (3, 18, 19).

This study aims to add to this important body of literature, in encouraging a random sample of child-bearing, rural Nigerian women from Cross River State to freely identify their health priorities, including physical, mental, emotional and social health and happiness. Women were encouraged to discuss health issues that were important to them. This study specifically considers the role of traditional customs and practices in the development of the research “working relationship” or “working environment” within which such participatory research can occur.

The objectives of this research are (1) to produce qualitative data on Cross River State women's self-identified health status and to discuss policy implications; (2) to explore the role of engagement in traditional customs to open the research working relationship.

### The value of health needs assessment with women

As part of a larger health needs assessment, we chose to do a separate study on women and their self-perceived health needs. We were very inspired by both the product and process of the needs assessment that we conducted using formal chieftaincy protocols. After several villages, however, we realized that using the official protocol meant that we were hearing mainly male voices. It seemed prudent to go straight to women and ask them about their priorities.

Visits to 12 villages in the state yielded a great deal of valuable information. Participants discussed needs for closer and cleaner water sources. In some villages the research team accompanied women and children on their daily walk for water, often several kilometers. Water supplies were often unsafe yet were the only available sources. Collection of rainwater was one possible solution. Sanitation was also named as another priority as was improved road transportation possibilities through the creation of more roads and public transportation methods. The need for continuous sources of nutrition was also identified and participants mentioned inland fish farming as a possible solution.

The need for water, sanitation, nutrition and transportation has been well documented in other health studies (20). It is unfortunate that these problems persist for it is certainly not for lack of knowledge, people power or motivation to address them. A complex matrix of structural power issues based in local and international relations is responsible for the continuation of the conditions of poverty in these villages.

## Methods

### Cultural research protocols

This study was part of a larger health needs assessment, which the author was directing. Working for a Canadian NGO that employed mainly Nigerians, she coordinated a large-scale health needs and asset-mapping exercise in Cross River State using Participatory Action Research (PAR) approaches. PAR was conceptualized by Orlando Fals Borda (21) and elaborated upon by others, including Patricia McGuire, who put women at the center of PAR.

Following traditional protocols, the team, composed of 4 Nigerians and 1 Canadian, requested a meeting with the Chief. In all cases the Chief accepted the invitation. In every instance, when the team arrived at the village, the Chief, accompanied by many others, greeted them warmly. Approximately 20–50 people welcomed the researchers. First the community would pray and offer libation to the ancestors. Next came dance; in this region many of the dances involved women only, so the author, being the only female on the research team, happily danced. After the celebratory dance it was time to feast. A wonderful meal of sumptuous traditional cassava, vegetables and meat were served and friendly conversation flowed. After these few hours of relationship building, the Chief would invite the researchers to speak about the reason they had come. The team explained that they worked for a Canadian non-governmental organization (NGO) that worked in community development and this project was focusing on health and they were visiting the village to ask people how best their village's health could be improved.

The cultural way in which the Chiefs of each village welcomed the research team was unparalleled in many work settings. A sophisticated understanding of human relationships being at the basis of productive work was built into the way in which the welcoming was done, through eating, feasting, dancing and informally relating to each other. Due to this warm participatory and highly sensory experience the research team was able to work much more effectively and efficiently.

This needs assessment process was an eye-opening and incredible way to conduct work, with the understanding that creating sound relationships are the basis for successful working collaborations. It was an outstanding illustration of PAR that communities led. In all cases the community identified the steps they wanted to follow, using traditional cultural norms to guide each step. Not only was the data gathering process thorough and comprehensive, it was done within context. For example, when village people told the researchers that sanitation was a concern they would lead the team to the sites they were using for waste disposal. From the visual and olfactory identification of holes in the ground that were clearly unhygienic, researchers were able to see the full size of the problem.

### Methodological approach

#### Conceptual rationale

This study was grounded in a community-based, qualitative approach aimed at understanding women's health-seeking behaviors in rural Nigeria. The methodological rationale was shaped by concerns about the dehumanizing effects of top-down research approaches, which have been criticized for being disrespectful- especially when used by authority figures to make decisions for those with less socioeconomic power (23). These methods may result in data that is not valid, as questioning from researchers perceived as superior can lead respondents to withhold or skew their answers. To mitigate this, we employed local women from the same villages, fluent in both Patois (Pidgin English) and local languages, to conduct respectful and culturally sensitive interviews.

### Participant recruitment and sampling

Interviews were conducted face-to-face with randomly selected participants between the ages of 18 and 45 years. The sample consisted of 70 women from 12 villages in 6 different local government areas. Additionally, we interviewed 15 traditional healers to enhance the statements made by our participants regarding their health-seeking behaviors.

### Data collection

Interviewers used culturally specific greetings and informal chatting in the local language or Patois to build rapport. To reduce discomfort for participants who could not read, interviewers filled out surveys on their behalf. The interviews were conducted in private settings, usually in participants' homes, creating a more conducive environment for open discussion.

The survey was developed by the author with feedback from Nigerian colleagues based at universities and NGOs. It was pre-tested with rural women and community educators from rural areas; several questions were revised for clarity and sensitivity. The question “What level of schooling do you have?”, for example, was changed to “Did you go to school?”. If the answer was positive, the level of schooling was inquired. Many participants had not attended a great deal of formal schooling, consistent with other rural Nigerian women (22), but had received extensive informal education in farming, sewing, and other areas.

Local women hired and trained as interviewers carried out data collection. They lived in the same communities as participants, which catalyzed trust and minimized perceived power imbalances.

### Literature review related to traditional medicine

To further contextualize participants’ experiences, particularly with respect to barriers in accessing formal health care, we did a literature review that included national legislative and regulatory documents related to traditional medicine. This review was conducted in parallel with the primary data collection and was used to inform the analysis presented in the “Efficacy and Regulation of Traditional Medicine” section. The literature offers relevant structural context for understanding participants' reported challenges.

### Data analysis and thematic coding

The data collected through in-depth interviews and surveys were analyzed thematically. We used the Framework Method to identify recurring patterns in the women's responses, drawing on their lived experiences to guide the structure of our findings. Notes and transcripts were reviewed manually and coded for major themes and sub-themes that emerged across multiple interviews.

The primary themes identified included:

• Socio-demographic patterns and fertility expectations
• Environmental and hygiene-related challenges
• Nutrition and food security
• Physical health concerns
• Health seeking behaviors and reliance on traditional medicine
• Perceived efficacy and accessibility of traditional vs. allopathic health systems
• Psychological health concerns

Within each theme, sub-themes were also noted. Under health-seeking behaviors, for instance, sub-themes included cost, proximity, cultural congruence with traditional healers, and dissatisfaction with formal health services. Under psychological health, sub-themes included sources of stress, definitions of happiness, and expressions of resilience.

These thematic groupings informed the structure of the integrated Results/Discussion section, which situates participant narratives within broader socio-cultural and health policy contexts. This approach also helped to highlight the interconnectedness of environmental, economic, and emotional factors in shaping women's health experiences.

### Ethical considerations

Despite these efforts, some participants expressed discomfort with certain questions- particularly those regarding nutritional status and children they had lost. One woman refused to participate after having previously taken part in a study in which her child died shortly after the interview. Many community members also expressed frustration and disappointment that research is often not followed by tangible improvements or implemented change, creating skepticism toward outside researchers.

## Results/discussion

### Socio-demographic data

The level of formal education of most respondents was fairly low. Sixty percent of them had not attended school while 30% had attended primary school and 10% had attended secondary school. The reason for this is two-fold. Their parents' income was not sufficient to enable all children to attend school. Like in other parts of the country, boys' education was a slightly higher priority than that of girls (24).

On average, each woman had given birth to six children, consistent with the national rural average (42), of whom only four survived. In many cases, their husbands had children from other women or wives, so the participants were often responsible for nurturing more than their biological children.

Forty percent of those interviewed did not know how many children they intended to have; of the sixty percent who did know, half of these wanted between six and eight children, and the other half wanted more than nine. Children are considered a blessing and in many cases, a means of social security, particularly in old age. The government has often encouraged Nigerians to rely on the family system for economic support, as the former provides minimal pension and welfare schemes.

Moreover, Nigerian society values a large number of children who have been successful and achieved educational, financial and other socially accepted markers of success. In all income brackets, a large number of children, particularly sons, are considered a symbol of social success. These attitudes are slowly changing, urged by the pressing economic conditions which force families to decrease in size as parents cannot afford to provide for a large number of children.

This study found that farming yam, cassava and rice is the largest source of income for survey participants. Women have their own farms and most of their yield is used to feed their families.

### Environment and hygiene

All women fetched their water from springs or streams and some villages had both sources. Women and children in these areas trek up to five kilometers to reach the water source, which often may be contaminated with pollutants or infested with worms that cause illnesses such as schistosomiasis. Some of the water is actually indigo/black in color and live organisms are visible with the naked eye. In many cases, one stream is used by three or four villages and the defecation and waste matter of one village flows into the drinking water source of another village. Even in villages where some inhabitants exclusively used one stream, people were fetching drinking water downstream, while others were washing and defecating upstream.

Many women and children spent up to six hours at the stream or spring, waiting in line, particularly at the end of dry season when water flows slowly. Water is therefore a major concern, if not the most critical basic need of these villages. Women and children suffer most as they fetch water and would stand to benefit most from boreholes, pumps, wells, stream purification and pipe systems. Sanitation is related to the problem of water. Over 82% of the women in this sample reported defecating and urinating in bushes. Approximately 18% claimed to use pit latrines.

Eighty-six percent of respondents deposit their household waste in a community garbage pile, which is burned after it accumulates to a certain size. The others burned their own. All in all, most refuse is recycled effectively, as goats and other animals consume much of the produce. Most people cannot afford to buy food with a great deal of packaging, so villages tend to be somewhat cleaner than urban centers.

Most cities and towns are fairly clean, however. National Environmental Sanitation Day occurs once every month. People are prohibited from being on the streets before 10 am on that day so that they stay at home to clean their compounds inside and out. Soldiers and police randomly check compounds to enforce the compulsory tidying and cleaning.

### Nutrition and eating habits

All women reported breastfeeding their babies. Baby milk formula is available but its prohibitive cost saves most village mothers from the numerous nutritional and economic problems associated with it, such as mixing the formula with contaminated water and spending 50% of family income on it.

Another positive finding was the lack of food taboos for pregnant women and children. Unfortunately, however, malnutrition is rampant in this state. The diet consists of mostly yam and cassava, with some rice. Protein foods such as beans, meat, fish, legumes and vegetables are considered a luxury when money is available to purchase these from the market.

Many families are unable to provide enough food for all their members from farm produce. Many village inhabitants confirmed this when discussing food scarcity in an abstract manner, however individual respondents claimed that there was always enough food to feed their family. Individual, family and community pride is extremely important. Not surprisingly, many people are reluctant to admit that they are not managing. Ninety percent of the participants claimed that they always had enough food for their families, although many of the women appeared to lack adequate nourishment.

Cross River State's economy is largely based on agriculture; banana, palm oil and fish are in surplus and sent to other parts of the nation, while palm kernel and cocoa are international export items. Therefore, it is worth examining the malnutrition that continues to exist in the state.

Cross River, as the name suggests, has rivers whose deltas are rich in fertile soil. In former times, malnutrition and lack of food were virtually non-existent. As in those times, fathers divide their land into parcels for the sons to inherit. Each parcel of land was sufficient to provide for the sons' families in former times. Wives were given a separate parcel of land which was usually used to feed the family. The husbands' land was usually used to produce crops for sale. This practice continues today and wealthy men often marry more than one wife so that they can have more land on their farms. However, as the population increases, each son receives a smaller plot of land. The former practice of leaving the land to lie fallow for a season in order for it to rejuvenate has been abandoned because of the growing need for food. This makes the soil less productive and compounds the effects of desertification and soil erosion.

Slash and burn farming techniques were efficient in the past, as the burning was thought to help fertilize the soil, after allowing it to lay fallow for some time. Again, without this fallow period, the soil suffers and so does farm yield. An additional problem is pests such as white ants, which have destroyed crops in one village in the catchment area of this survey.

### Physical health

Participants told us that people commonly suffered from malaria, diarrhea, anemia, hernia, waist pains, cough, eye problems and continuous headaches. Malaria is particularly a severe problem in Nigeria with the world's largest burden of this disease (25).

Participants stated that they usually visited traditional healers to treat these conditions although some claimed to also visit clinics and health centers. Purchasing imported drugs from local “hawkers” was also reported. Out of pocket expenses for healthcare particularly pharmaceuticals represent a great financial burden on families and illness often increases poverty levels (26). Often, when traditional healers' remedies were unsuccessful, our participants reported turning to allopathic medicine.

Women told us that they preferred to go to traditional healers because the cultural worldviews of these practitioners conformed to their own. They also told us that there were two other reasons that informed their decisions: (1) Cost: a typical treatment by a traditional healer costs a relatively small amount although this cost may vary greatly, and some traditional healers may require goats, chickens and large quantities of palm wine or local gin. (2) Geographical proximity: traditional healers are almost always located within the village; traveling time is thus less than health centers which are often far away, requiring time and transport costs. Furthermore, traditional medical practitioners far outnumber practitioners in other systems of medicine (27), so the likelihood of accessing traditional medical practitioners, particularly on short notice, is much higher.

Traditional healers, upon interviewing many of the respected ones in the catchment area, proved to be very open about the illnesses they felt they could treat and only encouraged patients who were afflicted with these. They were also ready to admit that their success rate is not 100%, attributing the failures to delayed arrival of patients or ancestral spirits. Ancestor worship is quite commonly practiced within many of the villages canvassed, despite the stronghold of the Christian church.

The first choice of care for approximately 80% of Africans are traditional healers (27), primarily because people believe in the efficacy of their systems of health care (29). PROMETRA, a West-African organization, composed primarily of West African medical practitioners, is working to integrate traditional medicine into ministries of health. Research on medicinal plants for the treatment of various infectious conditions is another part of PROMETRA's agenda. Traditional healers consult people for a variety of conditions including diabetes, infertility, mental health imbalances, TB, HIV, sickle cell, rheumatic concerns, asthma, migraines, allergies, Chlamydia, blood disorders, typhoid and stroke (28). Traditional healers, in particular parts of the continent, are renowned for their ability to cure certain ailments; for example, some specialize in healing wounds from snake bites while others are adept at healing broken bones. Psychotherapy, hydrotherapy and maternity care are widely practiced by Nigerian traditional healthcare providers and standardizing and regulating their practices would be an effective way of meeting the primary healthcare needs of the people whom they serve (31).

### Psychological health

All interviewees stated that they were unhappy at times (as most people everywhere are). Their reasons included illness of their family or themselves, death of children, lack of funds to cater to their family's needs, misunderstandings with husbands and physical and verbal abuse by the husband. For them, being happy meant:

“solving my problems”

“having a free mind”

“having money to cater for my children”

“having food for my family”

“not being sick and having less problems”

“when me and my children are healthy; when money is available to solve problems in the family”

It is clear that the economic and social situation of our participants strongly impacts their mental well-being. The stresses of poverty and patriarchy are particularly heavy burdens. Some of our participants seemed resigned to the difficulties they faced due to their status as working class women as the possibility for change seemed quite dim.

Nigerians have been identified as some of the happiest people in the world (36, 37). Despite the serious difficulties presented by poverty and social hierarchies, the women we interviewed virtually always smiled as they were working, walking, conversing or going about their daily affairs. Even the saddest of events, death, is punctuated by a celebration by that person's life. Nigerian funerals, like many other African funerals, often last for 3 days and involve feasting, dancing and singing, which are integrated with crying and collective grieving processes. Depression, anxiety and other mental health disorders, which are almost at pandemic levels in other countries (35) are prevalent in Africa mainly in situations of war, genocide, famine and other hardships (38). Otherwise, Nigerians' collective mental health status is exceptionally high particularly given the tremendous socioeconomic and political difficulties faced by most Nigerians on a daily basis (34).

### Efficacy and regulation of traditional medicine

The National Association of Traditional Medicine Practitioners (NANTMP) has lobbied the Nigerian government for many years to pass the Traditional Medicine Practitioner Bill (29). Like in many countries, the efforts of traditional medicine advocates striving for legitimacy have been met with fierce resistance. These struggles for medical dominance demonstrate the centrality of ideology and finances; power politics in medicine are partly about gaining access to legitimacy as a means of economic prosperity. Often these debates are couched in scientific language, masking underlying socioeconomic agendas (30). South African traditional medical practitioners faced similar challenges in passing legislation.

In South Africa, steps were taken in 2004 to legalize traditional medicine through the Traditional Health Practitioners Bill which was to regulate four kinds of practitioners: diviners (sangomas), herbalists (izinyangas), traditional birth attendants, and traditional surgeons (iingcibi) (34). Perhaps related to ideological and economic issues, this act was repealed on the basis of its constitutionality and apparent lack of public consultation. Further legal action was taken by a traditional healers’ association on the constitutionality of a witchcraft bill dating back to 1957. Legal battles ensued between associations of traditional healers and Doctors for Life International (DLI); DLI brings together various practitioners who uphold the following beliefs: “the sanctity of life from conception till death; sound science in the medical profession; and a basic Christian ethic in the medical profession.” (32)

Finally, in 2007/2008, the South African Parliament passed the Traditional Health Practitioners' Bill, which aimed to create a “regulatory framework to ensure the efficacy, safety and quality of traditional health care services; to provide for the management and control over the registration, training and conduct of practitioners, students and specified categories in the traditional health practitioners profession; and to provide for matters connected therewith.” (33)

It took until 2011 for nominations to the governing body to be sought and to date very little action has been taken. The reason that we have described these legislative efforts to regulate traditional medicine in South Africa in such detail is because they are reflective of the difficulties that exist throughout the continent and elsewhere.

### Recommendations

The results of the survey indicate that women participants are remarkably robust given their extremely difficult situations. They live in a poverty-stricken region within a lesser-industrialized nation. They therefore are vulnerable to regionalism, neo colonialism and patriarchy. Many are denied adequate education and sufficient means to sustain themselves.

Our interviewers informed us that most respondents appeared to be malnourished. This malnutrition is caused and compounded by overwork, lack of protein and iron, heavy reproductive demands, decreasing soil productivity, contaminated water supplies and other issues.

According to the women we interviewed, it appears that allopathic medicine is not the most prevalent form of healthcare. Clinics often charge too much money, are too far away and over-prescribe pharmaceuticals often motivated by profit. Traditional healers and birth attendants are the main health providers for this group of rural working class women, which is consistent throughout Nigeria (39). Our respondents told us their satisfaction rates with the care they receive from these providers are good to very good. No healthcare providers, however, are capable of curing the problems of poverty with medicine; social problems require social solutions. Many health problems in southeastern Nigeria are the result of colonial history and local hierarchies, which create gross inequities.

Priorities for lessening the gravity of this situation ought to be:

(a) Women's education which enables them to have more control over their environment—not colonial education which subtly teaches them to be subservient through adherence to the status quo. Literacy is a key component of this education. Studies in Kerala state in India have pinpointed female literacy as the key factor giving Kerala the highest standard of health in the country (40).
(b) Increasing the quality and amount of water is possible through wells, bore holes, stream purification and pipe-borne systems (41). This would dramatically reduce the time women spend collecting water, often under very difficult conditions and it would alleviate the illnesses created by contaminated water. Unfortunately, wasted effort has gone into several boreholes and wells, as accurate water table surveys are rarely conducted prior to drilling and digging. They are often dug when the water table is high. As a result, many boreholes and wells run dry after only a few months.
(c) Additional farming techniques beyond slash/burn methods, which may harm the land and destroy plants used by traditional healers. Different foods such as soya beans, lentils and quinoa would also serve to improve nutritional status as long as they are agriculturally viable and acceptable to people's palates.

### Future research directions

More research on African women's health status would help to elucidate how they survive and thrive in extremely challenging situations. Holistic analyses of spirituality, connections to the earth, musical expressions and other longstanding cultural traditions may explain the positive outlook held by African women even when they are facing difficulties.

This research and survey served as a useful training experience for local interviewers, helping them reflect on the relationship between poverty and ill health, and examine the benefits and drawbacks of conventional survey research. In future iterations of the study, we plan to incorporate participant observation and less obtrusive methods, which may yield more nuanced data and reduce participant fatigue or distress.

Further research on the effectiveness of traditional medicine would also help to enhance Nigerian women's health status. Moreover, research leading to the regulation of the practice of traditional medicine and protection of the public would also move this agenda forward. Lessons learned from South Africa would help to inform other African nations in their efforts towards regulation of traditional medicines.

## Conclusion

Women's health will not be improved until all aspects of their work are socially valued and they achieve greater autonomy and self-determination in their domestic, productive, reproductive and sexual lives. This is true for the participants in the survey as well as women in other parts of the world. Women participants said they birthed an average of 6 kids, but only 4 survived. The gravity of losing a child as well as the physical toll on their bodies cannot be overstated in emotional, social or economic terms. Traditional healers were likely quite successful in assisting women to heal emotionally and spiritually in light of high infant mortality rates, violence perpetrated by some of their husbands, the arduous walk to fetch water and the unhygienic conditions in which they relieve themselves every day.

Our participants displayed tremendous resilience in the face of great physical and socioeconomic challenges. They remained optimistic about their own future and that of their children even when they had suffered great losses. Most of the time, while southeastern Nigerian women are cleaning, cooking, fetching water and caring for their children, they are smiling and quick to laugh. Singing and dancing are regular parts of life. These joyful expressions help to keep women (and men) positive and living in the moment. Nigerians are amongst the happiest people in the world (34). Their spiritual and socio-cultural practices give them a strong sense of well-being as well as fostering connections between people. Strong relationships between family and friends anchor people's positive worldviews. Business practices around the world would benefit tremendously from traditional chiefs' welcoming and introductory practices in workplace settings. Our study underscores the importance of valuing African women's contributions to the economic development of their societies and making their mental and physical health a priority for researchers, health practitioners and governments.

## Data Availability

The raw data supporting the conclusions of this article will be made available by the authors, without undue reservation.
